# A novel simulador for agile and graphical modeling of surface plasmon resonance based sensors

**DOI:** 10.1038/s41598-023-46115-x

**Published:** 2023-11-02

**Authors:** Julio C. M. Gomes, Leiva C. Oliveira

**Affiliations:** Graduate Program of Computer Science (PPgCC), Department of Computer Science, Universidade Federal Rural do Semi-Árido (UFERSA), 59625-900 Mossoró, RN Brazil

**Keywords:** Nanophotonics and plasmonics, Materials science

## Abstract

Surface plasmon resonance (SPR) sensor is a consolidated technology for analysis of biomolecular interaction, largely applied in biology and pharmaceutical research. The simulation of the surface plasmon optical excitation response is an important step in the development process of SPR based sensors. The structure, design and configuration of the desired sensor benefits from a previous simulated analyses of the generated responses, defining operational conditions and feasibility of the selected materials to composed the optical coupling layers. Here an online web-based SPR sensor’s simulator is presented. With a visual-oriented interface, enable drag & drop actions to easily and quickly model a variety of sensor arrangements. Presenting an embedded materials database for metals, glasses, 2D materials, nanoparticles, polymers, and custom substances, the simulator enables flexible configuration for sensors operating in angular and spectral modes, as well as localized SPR. The light propagation through the multilayer of materials is presented in terms of Fresnel coefficients, which are graphically displayed. The so-called SPR morphology parameters can be visualized. Moreover, sensor dynamic behavior could be knowledge by a Sensorgram simulation. Localized surface plasmon resonance (LSPR) in homogeneous and spherical nanoparticles is also present in the simulator. Simulated scenario’s in various configurations, designs and excitation were performed and compare with other simulator. The proposed simulator guarantees comparable results with low-code, agile, and intuitive flow of execution.

## Introduction

The optical sensors based on Surface Plasmon Resonance (SPR)—an optical phenomenon resulting from photons and plasmons interactions^1–3^—explore the characteristics of multilayer arrays of different materials to detect molecular interactions, such as antigen-antibody reactions. In the SPR sensor design, it is essential to specify the structure and configuration of the multilayer, once it is responsible for transducing the interaction to be detected^4^. The multilayer specification determines the conditions for sensor construction, and defines functional blocks that comprise a sensor such as optical, electronic, mechanical, and fluidic elements.

The multilayer array is usually constructed from thin layers of materials at nanometric scales (thin films), forming a metal/dielectric interface. The metallic film is deposited over optically transmissive material and the dielectric can be a gaseous, aqueous solution or thin film (substance or sample) in which the molecular interaction to be detected occurs^5^. The array structure can be of a single metallic layer or with multiple metal/dielectric interfaces, on surfaces with periodic geometry (diffraction gratings), prisms, optical fibers, or even nanoparticles in the so-called localized SPR (LSPR). In addition, technical specifications related to the excitation spectrum, type of light source, type of optical detector, dimensions, angles, and geometry of the optical path also determine the feasibility of using certain materials to compose the multilayer. Furthermore, the typical operating modes of an SPR sensor: angular mode and spectral mode^6^, constitute another impacting factor in the development, data processing, and operational adjustments of an SPR sensor.

The choice of materials that will composes the multilayer is fundamental to obtaining a good SPR response. The type of metal and optically transmissive materials will define the resonance conditions for a particular substance. In general, it is necessary to know the optical properties of each medium and how the light-matter interaction occurs when crossing the different interfaces. The definition of the intended configuration and design for the SPR excitation is also needed. The characterization/simulation of the SPR sensor response with the chosen materials must be performed to check in advanced the quality of the proposed sensor and under what conditions it should operate.

A diversity of surface plasmon simulators can be found elsewhere^7–16^. Briefly, examples like the WinSpall^7,8^, SimSPR^12^, PAME^10^ and SWSO^11^ offers simulations in terms of Fresnel equations^3^ for the surface plasmons excitation based on the attenuated total reflection method (ATR)^5^, in both Kretschmann^1^ and Otto^2^ so-called configurations. Surface plasmon excitation approaches for Localized-SPR with nanoparticle simulations can be also founded in PAME and in Scattport.org website^16^. For SPR based on grating the SPRinG simulator^15^ include heuristics like Monte Carlo search and Particle Swarm optimization to project the sensor parameters^11,14^. Application to simulate kinetic interaction curves based on sensorgram graphs is the goal of the SPR-Simulation tool^13^. Moreover, general propose software for FDTD (finite-difference time-domain) simulation like COMSOL^17^ allow solving electromagnetic field equations and then simulate light-matter interactions for the surface plasmons conditions^9,18,19^. A summary of the mentions simulation tools is present in Table 1. In general, the simulators present a reduce set of options, with a few possibilities of materials and low flexibility for the parameters adjustments. The simulators for Surface Plasmons (SP)-excitation in ATR method typically simulates only one mode: or the angular (AIM) or the wavelength (WIM) interrogations modes. Although PAME and COMSOL present great functionalities, their interfaces sometimes are not practical to build the multilayer stack, with execution flow not agile, or even demanded of programming insertion. These software can lead to low productivity and less interaction due to difficult communication between the different professionals involved in the sensor design. Features like field distribution and temperature effect on the generated SPR response are not presented. Besides that, solutions like COMSOL or MATLAB programming platform eventuality required license and costs.

**Table 1:** Example of surface plasmons resonance simulators its available functionalities and features.

Features/remarks	Simulators								
	WinSpall	SWSO	SimSPR	PAME	SPRinG	SPR-Simulation	scattport.org	COMSOL	**EasySPR**
Reflectivity	X	X	X	X	X	–	X	X	X
Trasmisivity	–	–	–	–	–	–	–	X	X
Sensitivity	–	X	X	–	X	X	–	–	X
Field distribution	–	–	–	–	–	–	–	X	X
Temperature effect	–	–	–	–	–	–	–	X	X
Otto configuration	X	X	X	X	–	–	–	X	X
Kretschmann configuration	X	X	–	X	–	–	–	X	X
Grating configuration	–	–	–	–	X	–	–	X	–
Localized-SPR (nanoparticles)	–	–	–	X	–	–	X	X	X
Visual Multilayer	–	–	–	X	–	–	–	X	X
AIM	X	X	X	X	X	–	–	X	X
WIM	–	–	–	X	–	–	X	X	X
Sensorgram	–	–	–	–	–	X	–	–	X
Reaction/ kinetics	–	–	–	-	–	X	–	X	–
Prism based design	X	X	X	–	–	–	–	X	X
Optical fiber based design	–	X	–	X	–	–	–	X	X
Web	–	X	–	–	X	-	X	–	X
Drag &drop (productivity)	–	-	–	–	–	–	–	–	X
Materials database	–	–	–	X	–	–	–	X	X
SPR Curve morphology	–	–	–	–	–	–	–	X	X
Data export	X	–	–	X	–	X	X	X	X

In this work, we present a visual oriented Web-based simulator for SPR sensor. Called EasySPR, its embedded the most useful features/remarks to simulate multilayers SP-excitation. A quick and intuitive interface allow a systematic study of SPR-sensors in several configurations and modes, to improve design, construction and optimization of this type of sensor. The simulator includes an optical properties database of several materials. The simulator options are willing in the webpage to closely reproduce the process of using a SPR sensor. Graphical responses and the main extracted parameters are provided by the EasySPR.

## Surface plasmons resonance simulation on EasySPR

The basic theory of the resonant optical excitation for the well-know surface plasmons are extensive report in literature. From the early works^1,2,20^ to the modern introduction of surface plasmons^3,21^, the unfolding SP-applications and reports of sensor design majority describe the excitation based on the ATR method. The condition for SP-excitation is achieved on the ATR method by varying the coupling conditions between the horizontal component of the incident p-polarized light beam ($k_{px}$) and surface plasmons wavevector ($k_{sp}$), in which resembles a longitudinal charge density wave that propagates along with the surface metal interface.

The propagation of the surface plasmons $k_{sp}$ is obtained through Maxwell’s equations, that takes into account the boundary conditions of the multilayer structure^5^ at the metal/dielectric interface. The value of $k_{sp}$ is an approximation for the oscillation of surface plasmons, expressed by left part of equation (1), with $\epsilon _m$ the metal complex optical function, $\epsilon _m$($\lambda$) = Re{$\epsilon _m(\lambda )$} + *i* Im{$\epsilon _m(\lambda )$}; $\epsilon _d$($\lambda$) the optical value of the dielectric, and $\lambda$ is the incoming light-source wavelength. The incoming light beam cross an optically transmitting dielectric material, e.g., a prism with electric optical constant $\epsilon _p$($\lambda$), before hitting the metal layer at angle $\theta _1$.

1 $$\begin{aligned} \overset{\underbrace{\frac{2\pi }{\lambda } \sqrt{\frac{\epsilon _m \epsilon _d}{\epsilon _m + \epsilon _d}}}\cong }{k_{sp}} \overset{\underbrace{\frac{2\pi }{\lambda }\sqrt{\epsilon _p}\sin (\theta _1)}}{k_{px}} \end{aligned}$$

Changing the coupling conditions illustrated in Eq. (1), it is possible to develop sensors with two basic operating modes: Angular Interrogation Mode (AIM—*Angular Interrogation Mode*) and Spectral Interrogation Mode (WIM—*Wavelength Interrogation) Mode*). In the AIM mode a fixed wavelength is employed, and the incident angle is varying until the resonance condition is reached. The WIM mode is constituted by a polychromatic light hitting the multilayer at fixed angle, and being used to measure the reflectivity as a function of the wavelength.

### Fresnel analysis

The light matter interaction on the ATR method could be easily computed in terms of the Fresnel Analyses (FA)^22^. The FA gives the reflection/transmission/absorption coefficients for a p-polarized light beam crossing the multilayer arrangement. The reflectance curve (*R*), is calculated from the square of the reflection coefficient (*r*). The reflection coefficient in AIM $r(\theta )$ or WIM $r(\lambda )$, is given by equation (3) for a multilayer system with *m* layer, where a total transfer matrix $(M_{tot})$ is computed in terms of each medium transfer matrix $(M_j)$, admittance $(q_j)$ and absorbance $(\beta _j)$. The transfer matrix describe the wave transfer from medium *j* to $j+1$^3^.

2 $$\begin{aligned} R(\theta ) = \begin{vmatrix} r(\theta ) \end{vmatrix}^2 \text { or } R(\lambda ) = \begin{vmatrix} r(\lambda ) \end{vmatrix}^2 \end{aligned}$$

3 $$\begin{aligned} \begin{matrix} r(\theta ) \text { or } r(\lambda ) = \frac{(M_{11} + M_{12}q_m)q_1 - (M_{21} + M_{22})q_m}{(M_{11} + M_{12}q_m)q_1 + (M_{21} + M_{22})q_m} \end{matrix} \end{aligned}$$

4 $$\begin{aligned} M_{tot} = \prod _{j = 2}^{m - 1} \begin{vmatrix} M_{11}&M_{12}\\ M_{21}&M_{22}\\ \end{vmatrix} \end{aligned}$$

5 $$\begin{aligned} M_j = \begin{vmatrix} \cos (\beta _j)&(i sen(\beta _j))/q_j\\ -iq_j sen(\beta _j)&\cos (\beta _j)\\ \end{vmatrix} \end{aligned}$$

6 $$\begin{aligned} \left\{ \begin{matrix} q_j = \sqrt{\epsilon {_j}^2 - \frac{(\epsilon _1 sen(\theta {_1}))^2}{\epsilon {_j}^2}} \\ \beta {_j} = \frac{2\pi }{\lambda } d_j \sqrt{\epsilon ^2{_j} - (\epsilon _1 sen(\theta {_1}))^2} \\ \end{matrix}\right. \end{aligned}$$

**Figure 1:**
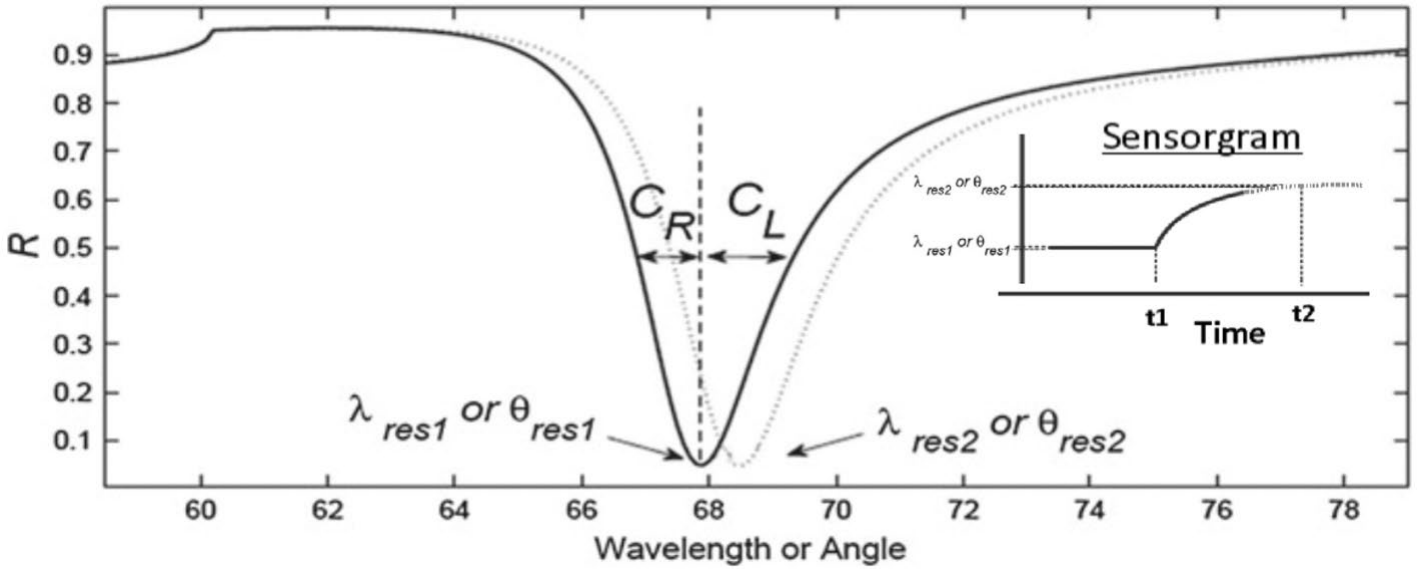
SPR curve example: reflectance vs. angle (AIM) or vs. wavelength (WIM). Resonance position indicated by the minimum value. The curve morphology parameters width ad asymmetry computed in terms of $C_R$ and $C_L$ values. Resonance position changes from $\lambda _{res1}$ to $\lambda _{res2}$ (WIM) or from $\theta _{res1}$ to $\theta _{res2}$ (AIM) as new substance is admitted in sensing area. Inset illustrated resonance time evolution, plotted in the so-called Sensorgram graph.

The graphic representation of $R(\theta )$ and $R(\lambda )$, called SPR curve (or reflectance curve), is typically employed to evaluate the sensor behavior. As present in Fig. 1, the resonance position is indicated by the minimum value of the curve. As new substance is sensing, the resonance condition changed and shifting the minimum value.

The curve morphology are also used for sensing^3^. In addition to the minimum position, the curve with and asymmetry are common used. The $C_R$ and $C_L$ distances taken at FWHM (*Full Width at Half Maximum*) are added to yield the width parameter ($C_R + C_L$), expressed in degrees or manometers for AIM or WIM respectively. The asymmetry is computed by the ratio of these distances ($C_R/C_L$). As scientific and technological tool for sensing molecular interaction, SPR sensors measure the refractive index changes of the substances under analyses mainly in terms of the SPR curve morphology changes. A sensorgram graph is used to shown the time evolution of the curve interested parameter as new substances are commuted.

#### Temperature effect

The SPR sensor response is affected by temperature fluctuations once the materials optical functions are temperature dependent. For the metal layer, Drude model is broadly employee to compute its temperature dependence. The metallic optical function $\epsilon _m$ can be rewritten as $\epsilon _m = 1- \omega _{p}^{2}/{\omega \left( \omega +i\omega _c \right) }$, where $\omega _{p}$ and $\omega _{c} = \omega _{ce} + \omega _{cp}$ represent the plasmon frequency and collision frequency of the metallic electron, both dependent of the temperature in the form^23–25^:

7 $$\begin{aligned} \omega _{p} (T) = \omega _{p} (T_0)\left[ 1+3\gamma _e \left( T-T_0 \right) ^{-1/2}\right] \end{aligned}$$

8 $$\begin{aligned} \omega _{ce} (T) = \frac{1}{6} \pi ^4 \frac{\Gamma \Delta ^*}{hE_F}\left[ \left( k_BT \right) ^2+\left( \frac{h\omega }{4\pi ^4} \right) \right] \end{aligned}$$

9 $$\begin{aligned} \omega _{cp} (T) = \omega _0 \left[ \frac{2}{5}+4\left( \frac{T}{T_D} \right) ^{5} \int _{0}^{T_D/T} \frac{z^4}{e^z -1}dz\right] \end{aligned}$$

where $\gamma _e$ refer to the thermal liner expansion coefficient; *T* is the temperature and $T_0$ the reference temperature; $\omega _{ce}$ and $\omega _{cp}$ are the contributions of electron-electron and phonon-electron scattering on $\omega _{c}$ respectively; $E_F$ Fermi energy; $\Gamma$ is the Fermi-surface average of scattering probability; $\Delta ^{*}$ is the fractional Umklapp scattering; $k_B$ Boltzmann constant; *h* Planck’s constant; $T_D$ Debye temperature; $\omega _0$ is a constant calculate for each specific metal. The temperature dependence of the metal film thickness can be modeled using the expression $d(T) = d_0 \left[ 1 + \gamma ^{'} \left( T-T_0 \right) \right]$^26^, with the appropriate thermal expansion coefficient $\gamma ^{'} = \gamma _e (1+\mu )/(1-\mu )$, where $\mu$ is a Poisson’s number, around $\approx$ 0.44 for the Au metal film^26^, resulting in $\gamma ^{'}$ twice as large as $\gamma _e$ and an increase of *d* in the order of 0.018 nm for 90$^\circ$C temperature changes versus $T_0$ .

For glasses materials, the refractive index ($n\approx \sqrt{\epsilon }$) variations with temperature could be estimated as $n(\lambda ,T)=n(\lambda ,T_0)+\Delta n(\lambda ,T)$, with $\Delta n(\lambda ,T)$ expressed by^27^:

10 $$\begin{aligned} \Delta n (\lambda ,T) = \frac{n^2\left( \lambda , T_0 \right) -1}{2n\left( \lambda , T_0 \right) }\left( D_0 +D_1\Delta T+D_2\Delta T^2+\frac{E_0+E_1\Delta T}{\lambda ^2 -\lambda _{0}^{2}}\right) \end{aligned}$$

where $\Delta T$ correspond to temperature difference versus $T_0$; and $D_0$, $D_1$, $D_2$, $E_0$, $E_1$, and $\lambda _0$ are constants depending on glass type. For BK7 glass and TOPAS polymer, these constants are valid for a wide temperature range, with *n* increases with temperature.

Likewise, the refractive index of liquid/gases substance varies as a function of temperature. Validated data and formulation for the refractive index of water and steam varying from -12$^\circ$C to 500$^\circ$C, wavelength range from 200 to 1100 nm, and a density range from 0 to 1060 kgm$^{-3}$, is expressed with Lorentz-Lorenz function as follow^28^:

11 $$\begin{aligned} \frac{n^2-1}{n^2+2}\left( \frac{1}{\bar{p}} \right) =a_0+a_1\bar{p}+a_2\bar{T}+a_3\bar{\lambda ^2}\bar{T}+\frac{a_4}{\bar{\lambda ^2}}+\frac{a_5}{\bar{\lambda ^2}-\bar{\lambda _{UV}^2}}+\frac{a_6}{\bar{\lambda ^2}-\bar{\lambda _{IR}^2}}+a_7\bar{p} \end{aligned}$$

where $\bar{p}$, $\bar{T}$ and ${\bar{\lambda }}$ are dimensionless variables in the form $p/p^*$, $T/T^*$ and $\lambda /\lambda ^*$ respectively, with $p^*$, $t^*$ and $\lambda ^*$ are reference constants; $a_0$ to $a_7$ are coefficients; $\bar{\lambda _{UV}}$ and $\bar{\lambda _{IR}}$ are constants. Extensive literature regards temperature effect on SPR sensor and optical properties of materials can be found elsewhere^23,26,29^.

### Mie analysis

Employing nanoparticles (NP) smaller than the wavelength of light and composed by the same conductive material, the surface plasmon will be localized at the NP, and thus represent an eigenmode of the wave equation. In general, the measurement of the extinction spectrum of a NP is used for sensing refractive index changes of a medium that surrounds the nanoparticle. The theoretical model used by the EasySPR for LSPR simulation, utilizes the Mie light scattering theory. The Mie theory applies to homogeneous and spherical nanoparticles. There are extensive literature about this electromagnetic model to explain the scattering ($c_{scat}$), extinction ($c_{ext}$) and absorption $(c_{abs} = c_{ext}-c_{scat})$ at NP resonance condition, solved by follows^3,30,31^:

12 $$\begin{aligned} c_{ext}=\frac{2\pi }{k^2}\sum _{\infty }^{l=1}(2l+1)Re(a_l+b_l) \end{aligned}$$

13 $$\begin{aligned} c_{scat}=\frac{2\pi }{k^2}\sum _{\infty }^{l=1}(2l+1)Re(a_l^2+b_l^2), \end{aligned}$$

in which for the same permeability of the NP and the dielectric surrounding, the scattering coefficients $a_l$ and $b_l$ can be computed from first ($\psi$) ans second ($\xi$) order Ricati-Bessel functions:

14 $$\begin{aligned} a_l = \frac{m \psi _l(mx) \psi _l^{'}(x)-\psi _l^{'}(mx) \psi _l(x) }{\psi _l(mx)\xi _l^{'}(x)-m\xi _l^{'}(mx) \psi _l^{'}(x)} \end{aligned}$$

15 $$\begin{aligned} b_l = \frac{\psi _l^{'}(mx) \psi _l^{'}(x)-\psi _l^{'}(mx) m\psi _l(x) }{\psi _l(mx)\xi _l^{'}(x)-m\xi _l(mx) \psi _l^{'}(mx)} \end{aligned}$$

where *k* is the wavevector of incident light; $m=n_{NP}/n_{sd}$ the relation between refractive index of NP ($n_{NP}$) and surrounding dielectric medium ($n_{sd}$); and $x=kr^{*}$, with $r^{*}$ representing the NP radius.

## EasySPR—simulation of surface plasmon resonance

The EasySPR was development based on MVC (Model, View, Controller) design pattern^32^. The use interface (View layer) is formed by HTML, CSS and JQuery markup languages and gives user interaction functionalities in a low-code strategy. The user information is passed to the Model layer in a JSON format (JavaScript Object Notation) for read/write the data. Ajax programming (Asynchronous JavaScript and XML) is used to deal with the markup languages and generate the interactive Webpage and graphical refresh by means of asynchronous calls to the server^33^. Then, the Ajax codes communicate with Ruby code (Controller layer)^34^ employed to realized calculation and other mathematical operations of the simulator. At the the controller, different modules are responsible for each simulation type (AIM, WIM, LSPR, and Sensorgram).

**Figure 2:**
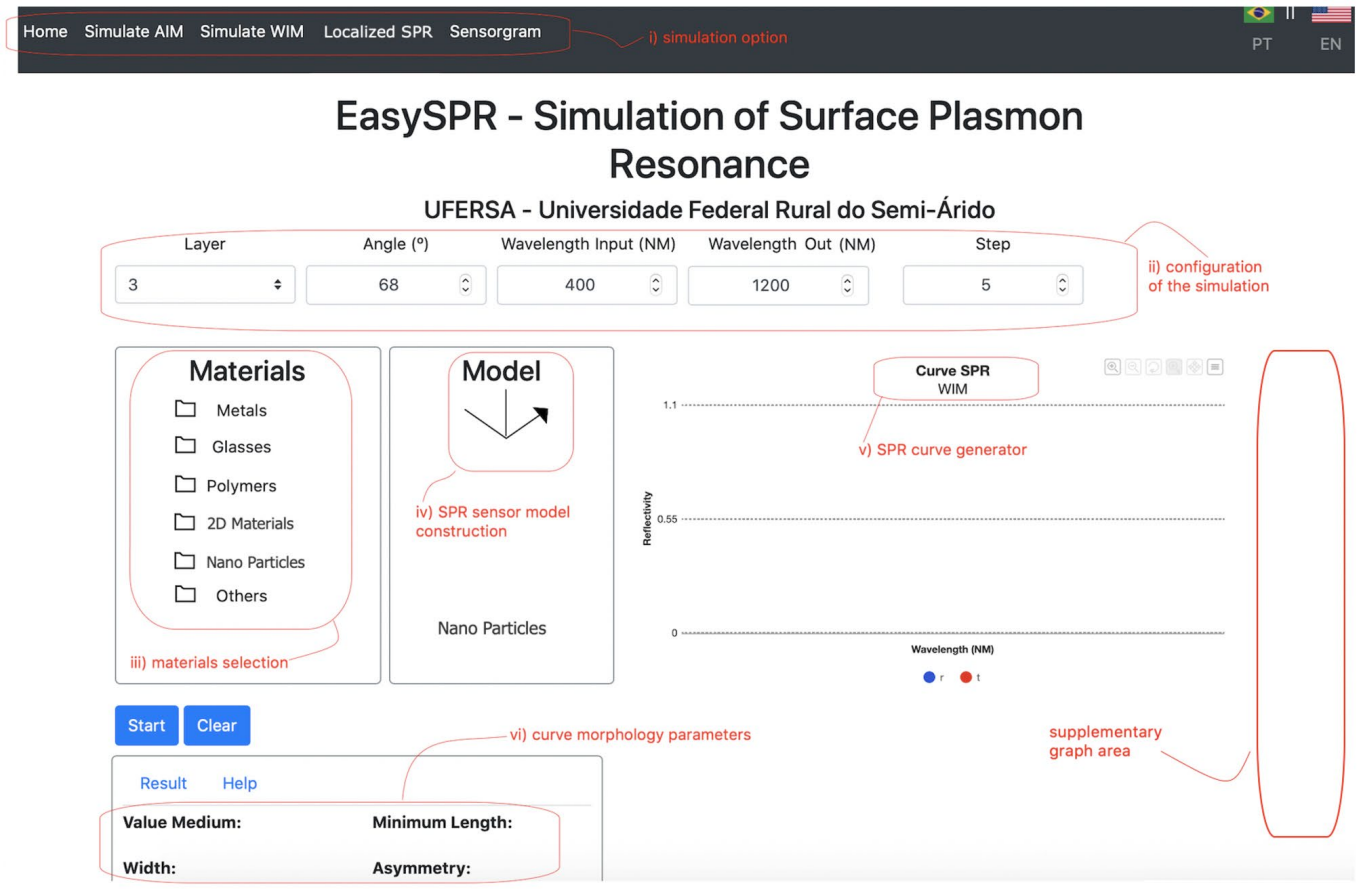
The EasySPR—simulation of surface plasmon resonance homepage. Logical parts of the simulator (i) simulation option, (ii) configuration of the simulation, (iii) materials selection, (iv) SPR model construction, (v) Graph/curve area and to (vi) morphology parameters are indicated.

The proposed web-based simulator is presented in Figure 2. It is composed by the follow parts: (1) simulation option: AIM, WIM, Localized SPR or Sensorgram simulation; (2) configuration of the simulation; (3) materials selection; (4) multilayer construction (SPR sensor model); (5) Graph/curve area generator (SPR curve, LSPR curve or Sensorgram), and (6) Curve morphology parameters.

The EasySPR simulator possibility the AIM and WIM simulation as well as LSPR simulation in wavelength mode and Sensorgram simulation in angular mode. For each chosen mode, it is necessary to define the number of layers (min. of 3 and max. of 10) present in the multilayer structure. Next, the configuration parameters for the chosen type of simulation must be set. The AIM requires the follow parameters: light source wavelength, the initial and final angles range, and angle resolution (step). The step parameter could be represent the set-up characteristic in terms of optical arrangement and camera resolution. For WIM mode, the initial and final wavelength range of the light source, its incident angle, and the spectrometer resolution (step parameter) must be defined.

**Figure 3:**
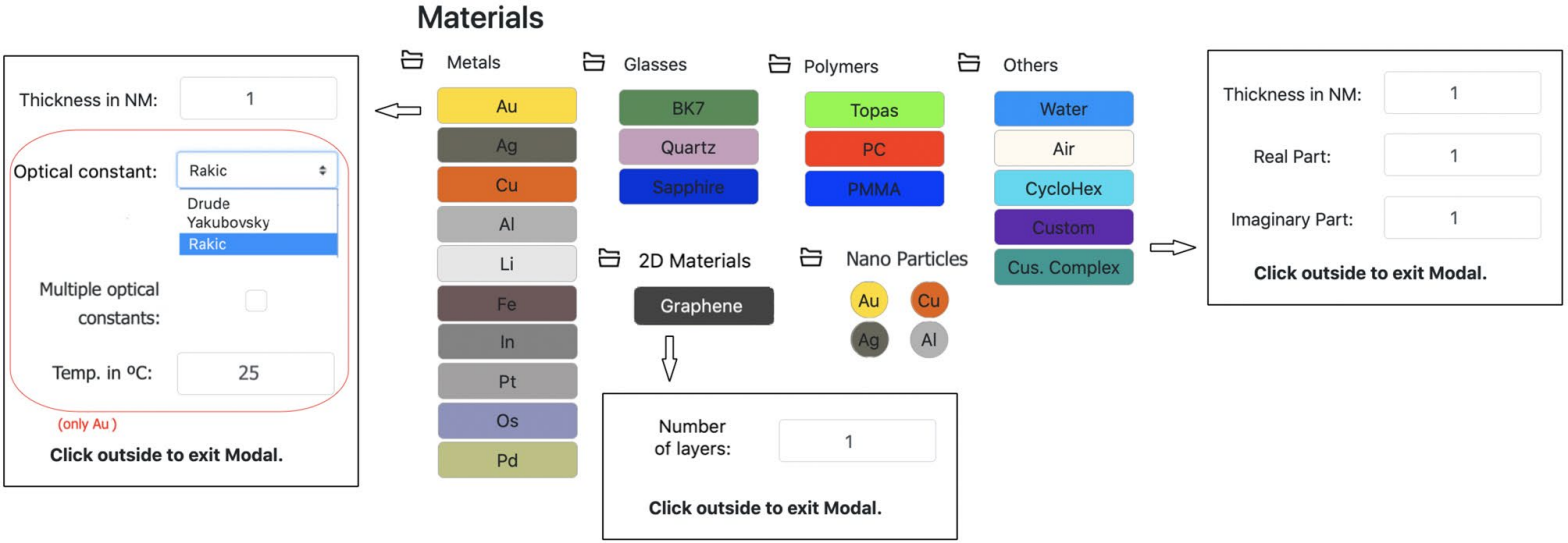
Database materials of the EasySPR simulator. Elements dived into six classes: Metals, Glasses, Polymers, 2D Materials, Nano Particles and Others. The thickness of the selected material could be adjusted by the user. Details for: (i) the custom materials option after double click on the graphical element: possibility to define thickness and (complex)refractive index values; (ii) the number of graphene layers could be set; (iii) for the Au metal different CDF values and the temperature effect simulation in the optical constants based on the Drude representation could be configured.

After defining the simulation parameters, the multilayer structure, here called SPR sensor model, must be created. To define the Model, the software has a Database containing the optical properties of the main Materials used in the manufacture of SPR sensors. The simulator’s database included different types of materials, which were previously analyzed in the SPR sensor context^3^. Figure 3 show the database elements. For metals there are noble metals, transitions metals of the platinum group, common transition metals and other common metals. The actual list is composed by gold (Au), silver (Ag), copper (Cu), aluminum (Al), lithium (Li), iron (Fe), indium (In), platinum (Pt), osmiun (Os) and palladium (Pd). For dielectric materials, the database contains solid glasses (BK7, Quartz and Sapphire), polymers (TOPAS, PC and PMMA), liquid dielectrics (Water and cyclo-Hexane) and Air. Graphene is present as a two-dimensional material, and nanoparticle of noble metals are available. The thickness of the selected material could be adjusted by the user. To improve the materials lists and customize the simulation, the so-called Custom materials are included. It allows the user informs the desired (complex)refractive index values of a layer that is composing the Model. It is important to point out that the experimental data for the metal complex dielectric/optical function (CDF) and optical dispersion (OD) for dielectrics were adjusted as a function of wavelength using a interpolation. This procedure allows greater reliability of the responses, as well as greater flexibility to explore new wavelengths/angle for optical couple condition. Furthermore, for Au different representations of CDF and the temperature of the simulation can be adjusted.

**Figure 4:**
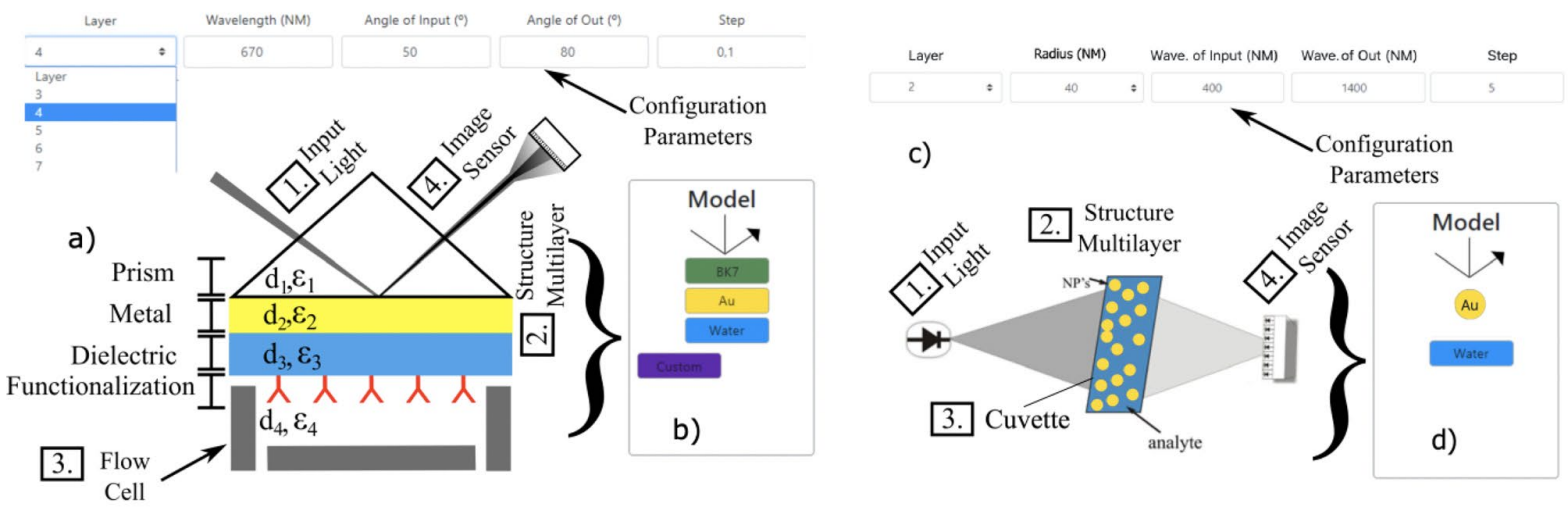
(**a**) The multilayer system excited with a p-polarized light for a SPR sensor. Example of a four layer multilayer structure. All elements are indicated. (**b**) Conceptual idea representation of the Model construction on the proposed EasySPR simulator. (**c**) LSPR multilayer approach utilizing nanoparticles (NP’s) immersed in a surround analyte, contained in a glass vessel; all optical components are indicated. (**d**) Conceptual representation of LSPR Model.

### Multilayer construction

To build a sensor and simulate the phenomenon, the multilayer structure is created for the sensor model by drag &drop materials on the Model area. The sensor model concept is illustrate in Fig. 4. For the traditional SPR, the model is built in top-down approach, e.g., the light hitting the first material included and the propagation follow the subsequent elements. As illustrated in the figure, an example of sensor lattice composed by BK7 Prism, gold metal deposited on it, at liquid interface (water element) content an analyte substance (Custom element) is represented by the four layers added on the Model area.

**Figure 5:**
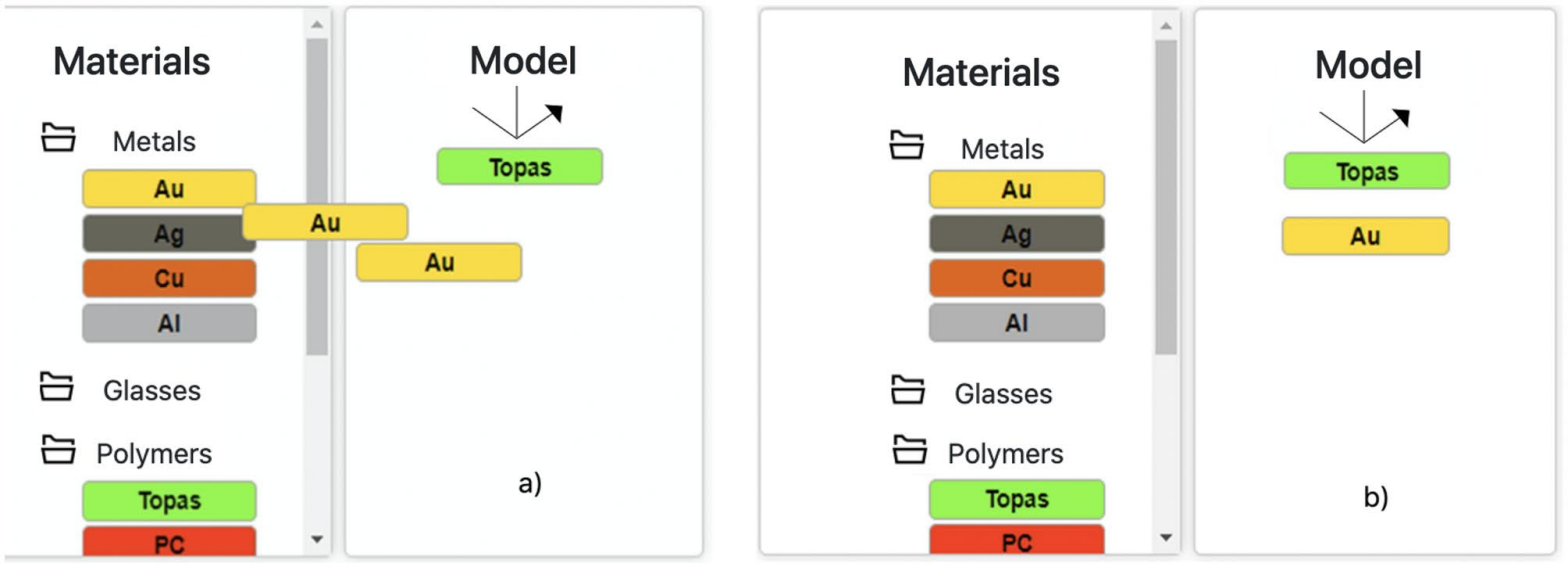
Proceedings to create the SPR sensor model. Materials are added in the Mode area. First Topas element is added (1st layer) follow by Au (2nd layer). (**a**) Dragging element to the model area. (**b**) Element dropped.

In terms of software solution, each material is a drag &drop visual element. Thus, a substance is moved and placed at the model area (see Fig. 5) in a intuitive and practical way. By clicking, a modal window is open for the user adjust the thickness of the material, the refractive index values (for the Custom materials) or other available parameter.

**Figure 6:**
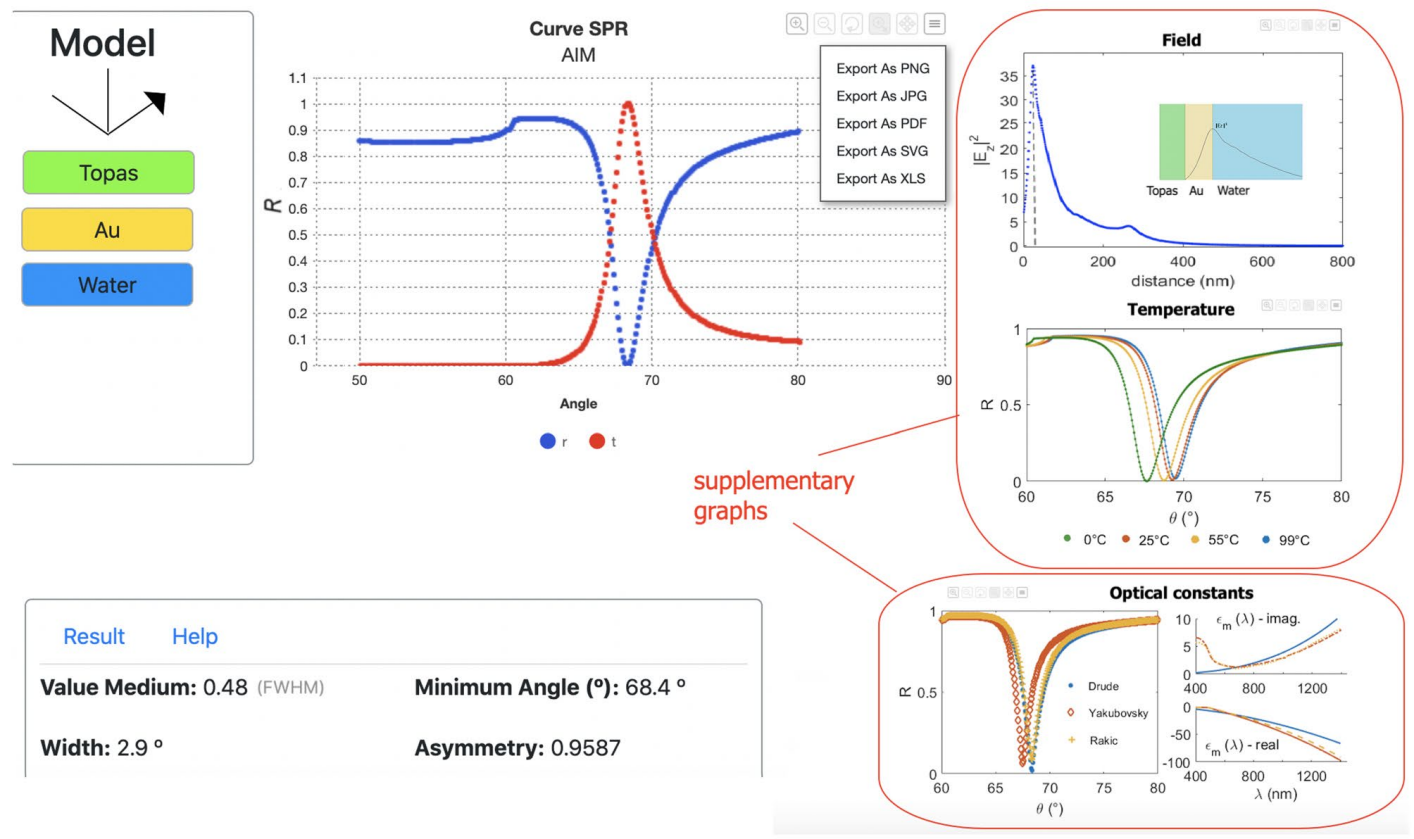
Simulation results for a sensor model Topas/Au/Water. Curves and morphological parameters are presented. Details for supplementary graphs and the data exports options.

Once the model is created, by pressing the Start button the SPR response is calculated and shown, as indicated in Fig. 6. The graphical area is destined to display the SPR curve according to the user selection in the legend-buttons “Data Selector”. The transmittance curve $$T(\theta ) = \begin{vmatrix} t(\theta ) \end{vmatrix}^2$$ or $$T(\lambda ) = \begin{vmatrix} t(\lambda ) \end{vmatrix}^2$$ from Fresnel reflection coefficient in AIM $t(\theta )$ or WIM $t(\lambda )$, computed as: $$\begin{matrix} t(\theta ) \text { or } t(\lambda ) = \frac{2q_m}{(M_{11} + M_{12}q_m)q_1 + (M_{21} + M_{22})q_m} \end{matrix}$$, is also possible to visualized. The morphological parameters asymmetry, width, resonance position (curve minimum value: $\theta _{res}$ for AIM or $\lambda _{res}$ for WIM), and medium value of the curve (FWHM position) are extracted from the SPR curve, and exhibited below the graphical area. For Au film, three supplementary graphs are also displayed: (1) the intensity of the electromagnetic field distribution, shown that decays exponentially with distance normal from the analyte; (2) the SPR curves for different temperatures, as example of one temperature lower and two higher from the set simulated value of 25$^\circ$C; and iii) the impact of different CDF values on the SPR curve. The available optical constants are based on Rakić^35^ and Yakubosky^36^ dataset as well as on the Drude model. Furthermore, the simulated data can be saved/exported in different formats through the control buttons present in the graphic. The Clear button can be used to clean the model and displayed graphs.

**Figure 7:**
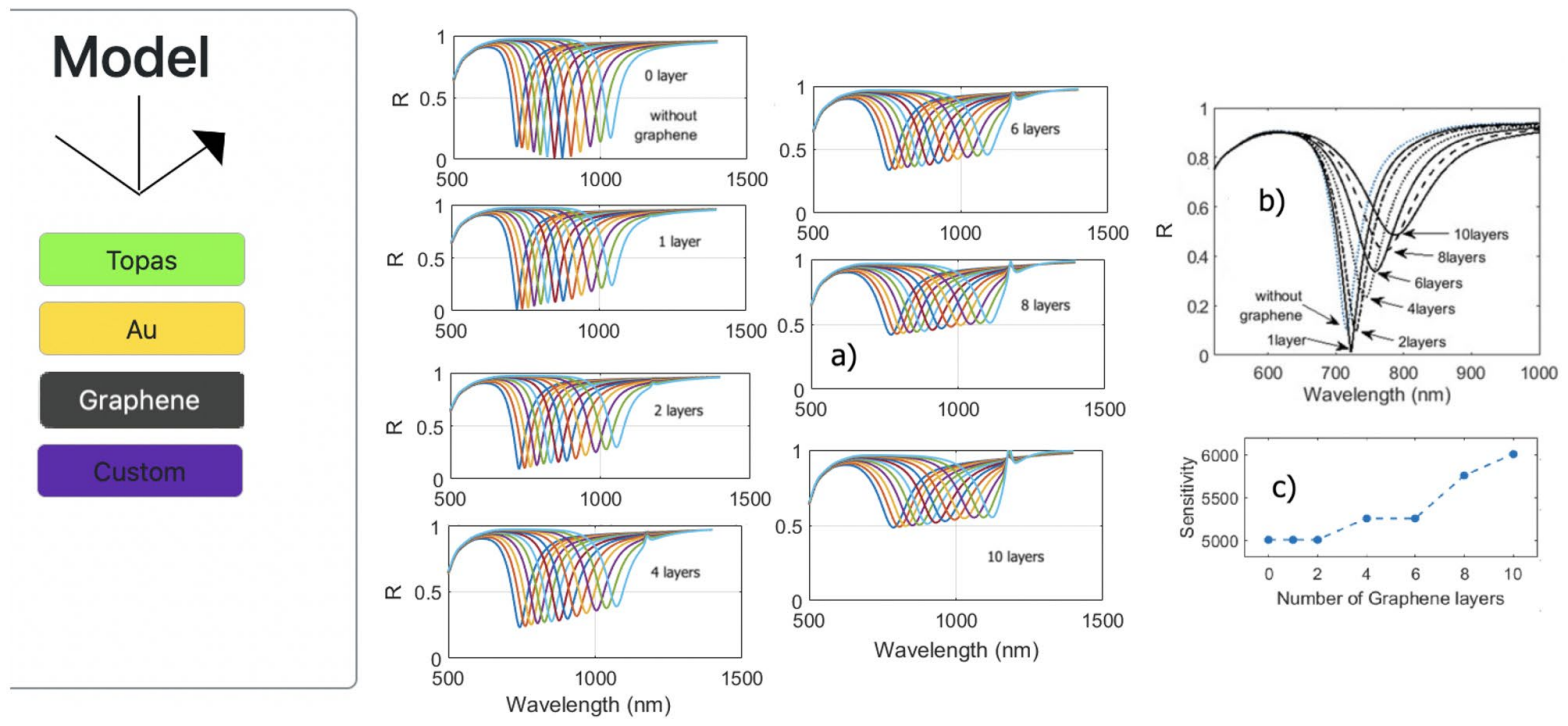
(**a**) Graphene SPR response simulation for various aqueous refractive index and numbers of graphene layers. (**b**) Summarized SPR curves for the selected amount of graphene layers. (**c**) Sensitivity versus number of graphene layers.

The EasySPR provide a 2D Material simulation with the graphene. The Fig. 7 presents the SPR curves for refractive index variation range 1.33 $< n_d<$1.39 (Custom Material) under WIM-conditions for different number of graphene layers. The nano-structure of a single layer of graphene is 0.34 nm thick^37^. The sensitivity increases with the amount of graphene layers (starting from 4 layers) but decrease the contrast/depth of the SPR curve. Graphene and other two-dimensional materials have attracted much attention because provides a high surface-to-volume ratio leading to higher sensitivity SPR sensors. The results are equivalent to related works identified in the literature^37–39^.

#### LSPR approach

**Figure 8:**
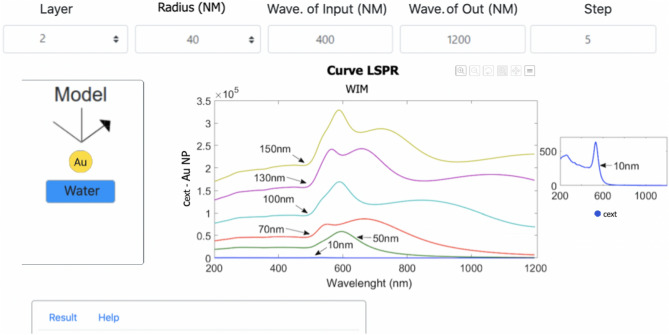
Simulation results of the extinction spectrum for a LSPR-sensor model Au-NP/Water for different particle sizes. The peak of absorption indicates the resonance position. Detail for the optical absorbance for Au-particles with 10 nm.

For the localized SPR, the model is composed by the metallic nanoparticle and the surrounding dielectric medium. The EasySPR simulates the optical absorption characteristics ($c_{ext}$) for spherical NP with a selected radius at wavelength interrogation mode. An example of Au-NP/Water is represented by the two layers added on the Model area, as indicated in Figure 8. The peak of absorption indicates the resonance position. The influence of NP shape^31^ and the uses of a corrected/compensated CDF values for nanoparticles^30^ are not included in the LSPR simulation.

### Sensorgram visualization

As another enlightening way to simulate a sensor behavior, the EasySPR present the temporal evolution of the resonance position for different substances: the sensorgram graph^40^. The sensorgram is simulated for a sensor operating at angular interrogation mode. The simulation is realized for a sensor model composed by three layers in the form: Substrate/ Metal/ Substance. Thus, the Model will include only two elements from the database: the substrate and the metal. The substances inlet/outlet are configured separately.

**Figure 9:**
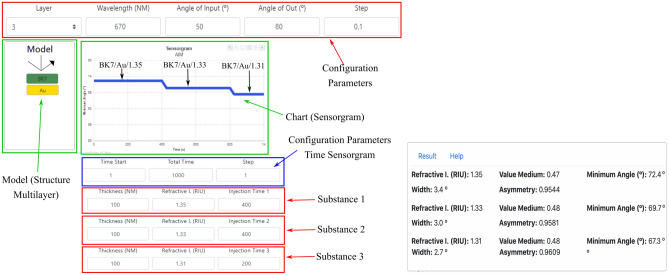
Sensorgram simulation option. Example for a multilayer composed by BK7 /Au / and substances sequenced with refractive index of 1.35, 1.33 and 1.31, injected at times ${t_0}$, ${t_{401}}$ and ${t_{801}}$ respectively.

The Fig. 9 present the sensorgram option. Initially, the configuration parameters must be set and the sensor model created. The sensorgram need temporal adjustment for each substance. The start time of the substances inlet, the total experiment duration, and time resolution (step) could be adjusted by the user. The sensorgram graph is illustrated with switching 3 substances, one each time. Thickness, refractive index (RI) and injection time is set. The injection time refers to the duration of the substance in contact with the sensing layer. The Fig. 9 shows a sensorgram for the multilayer BK7/ Au (50 nm)/ Substance 1: (thickness = 100 nm, RI=1.35, and Injection time = 400 s); follow by BK7/Au (50 nm)/ Substance 2: (thickness = 100 nm, RI=1.33, and Injection time = 400 s); and BK7/ Au (50 nm)/ Substance 3: (thickness = 100 nm, RI = 1.31, and Injection time = 200 s).

The resonance position of the SPR curve for each substance is displayed at the sensorgram, simulating the sensor behavior. The results is a time series in which from 1s to 400s the sensor measurement the Substance 1, with $\theta _{res1}$ = 72.4$^\circ$; from 401s to 800s the Substance 2, with $\theta _{res2}$ = 69.7$^\circ$; and beginning at 801s until 1000s the Substance 3 with resonance at $\theta _{res3}$ = 67.3$^\circ$. It is important to point out that surface coverage^41^, kinetics association and dissociation^42^ and noises^43^ are not yet included in the sensorgram simulation.

**Figure 10:**
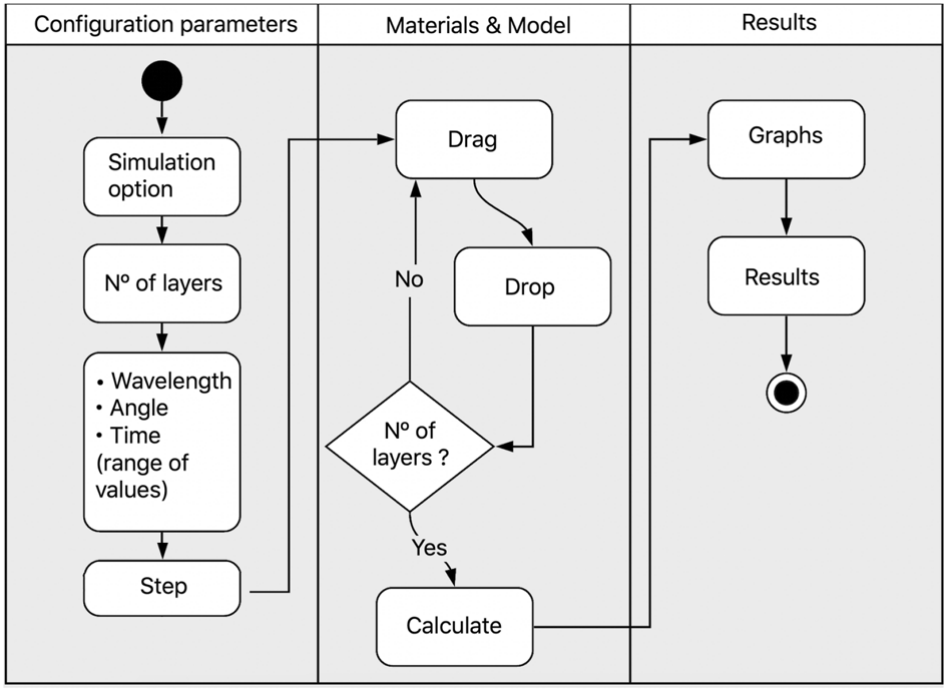
EasySPR flowchart. The web simulator enables the user to configure the simulation parameters, and create a sensor model by drag &drop friendly approach. The delivery results in graphs and numerical values could be used to guide SPR sensor construction.

In summary, the flowchart of the EasySPR Simulator proceedings is present in Fig. 10. In the beginning, the simulation option (AIM, WIM, Sensorgram) follow by the configuration parameters set are configured. Then, the sensor Model (multilayer structure) is created. And finishing, the results are present in graphs and numerical values.

## Results

To analyze and compare the EasySPR, different simulation scenarios’ were proposed/reproduced and the results were compare with other simulators. Five different scenarios were proposed as follow:

- Three scenarios in AIM with Otto configuration, previous reported in^21,44,45^. The simulators WinSpall^8^, Sim-SPR^12^, SWSO^11^, and COMSOL^9^ were compared.
- Two scenarios in WIM with Kretschmann configuration, reported in^46^ and^47^. The simulators SWSO^11^ and COMSOL^9^ were used.

The Table 2 summarized the multilayer for each scenario. For AIM scenarios, the angular spectrum was obtained for the range 30$^\circ$ $< \theta _i<$ 50$^\circ$. The first one consider a sensor with (1) BK7 prism ($n_p$), (2) 1000 nm Air gap and (3) thin gold layer with 50 nm. The light beam wavelength is 800 nm. The second used five layer in order to attest the metal layer thickness precision and Custom element EasySPR gadget. Thereby, the sensor model is composed by: (1) BK7 prism, (2) 300 nm Air gap, (3) 310 nm polyvinyl alcohol (PVA)—as another dielectric material by means of Custom element, (4) 50 nm gold layer and (5) BK7; being SP-excitation with 632.8 nm wavelength. The third scenario used a (1) BK7 prism, (2) a 2200 nm Air gap, and 50 nm layers of (3) quartz and (4) gold; 975.1 nm wavelength was used.

**Table 2:** Summarized main information of the scenarios used at simulation tests

Scenario 1							
Ref.^44^ (AIM)	Light source (fixed)	$n_p$		$n_{Au}$		$d_{Air}$	$d_{Au}$
	$\lambda =$ 800 nm	1.50		0.23+4.5i		1000 nm	50 nm
Scenario 2							
Ref.^45^ (AIM)	Light source (fixed)	$n_p$	$n_{\text {PVA}}$	$n_{Au}$	$d_{\text {PVA}}$	$d_{Air}$	$d_{Au}$
	$\lambda =$ 632.4 nm	1.515	1.5	0.172+3.440i	310 nm	300 nm	50 nm
Scenario 3							
Ref.^21^ (AIM)	Light source (fixed)	$n_p$	$n_{quartz}$	$n_{Au}$	$d_{quartz}$	$d_{Air}$	$d_{Au}$
	$\lambda =$ 975.1 nm	1.5079	1.4507	0.2151+6.2835i	50 nm	2200 nm	50 nm
Scenario 4							
Ref.^46^ (WIM)	Angle (fixed)	BK7	Cu	Au	$d_{Cu}$	$d_{Au}$	$d_{H_2O}$
	$\theta =$ 68$^\circ$				40 nm	5 nm	100 nm
Scenario 5							
Ref.^47^ (WIM)	Angle (fixed)	BK7	Al	$n_{Al_2O_3}$	$d_{Al}$	$d_{Al_2O_3}$	$d_{H_2O}$
	$\theta =$ 68$^\circ$			1.8127	20 nm	4 nm	100 nm

**Figure 11:**
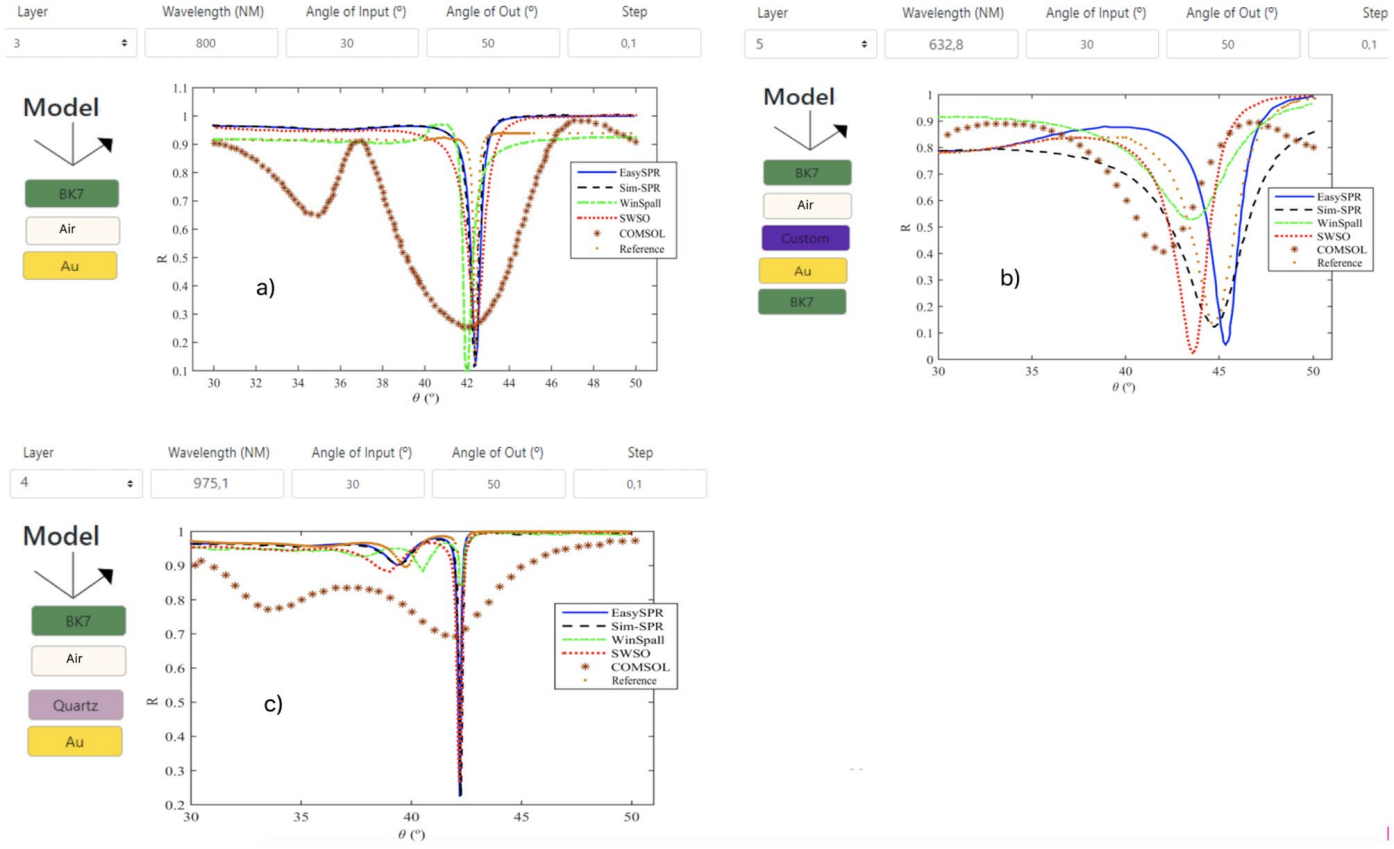
AIM SPR curves and respective models for the scenarios (**a**) 1, (**b**) 2 and (**c**) 3. Responses for the EasySPR and others simulators used for comparison are indicated.

The Fig. 11 illustrated the sensor models and respective SPR curves, generated by the simulators. The graph colors indication is EasySPR (blue solid line), Sim-SPR (black dashline), WinSpall (green dashline), SWSO (red dot line), COMSOL (star) and a Reference simulation (orange dots) from^3^. In the Fig. 11a, c is possible to note that the EasySPR curves present resonance angle position similar to the Sim-SPR, WinSpall and SWSO simulators in the scenarios 1 and 3. The curves are very sharp (low values of width and asymmetry) and with a prominent deep, in accordance to the reference curve. The COMSOL results from^9^ present a broadened curve, despite having similar $\theta _R$ position. The scenario 2 (Fig. 11b) present more differences between the curves. The EasySPR capture the essential shape of the reference curve, with a prominence around 40$^\circ$ and sharp dip. The Sim-SPR and SWSO also present equivalent responses, with the former one showing the minimum more closely to the reference with curve shape more distorted. The simulators COMSOL and WinSpall gives higher distorter curves in terms of the morphology parameters.

For WIM the scenarios reproduced the simulations early report in^46,47^. For a sensor in Kretschmann configuration, the first scenario^46^ consider (1) BK7 prism, (2) 40 nm thin cooper layer, follow by (3) 5 nm protective gold layer in (4) aqueous environment; input angle of 68$^\circ$ and light source range of 400 nm $< \lambda _i<$ 1200 nm. The second WIM scenario^47^ used a sensor with (1) BK7 prism, (2) 20 nm aluminum film plus a (3) thin oxi-layer Al$_2$O$_3$ of 4 nm (as a Custom element), and (4) H$_2$O interface; the simulation performed with a broadband light source with 200 nm $< \lambda _i<$ 1200 nm and input angle of 68$^\circ$.

**Figure 12:**
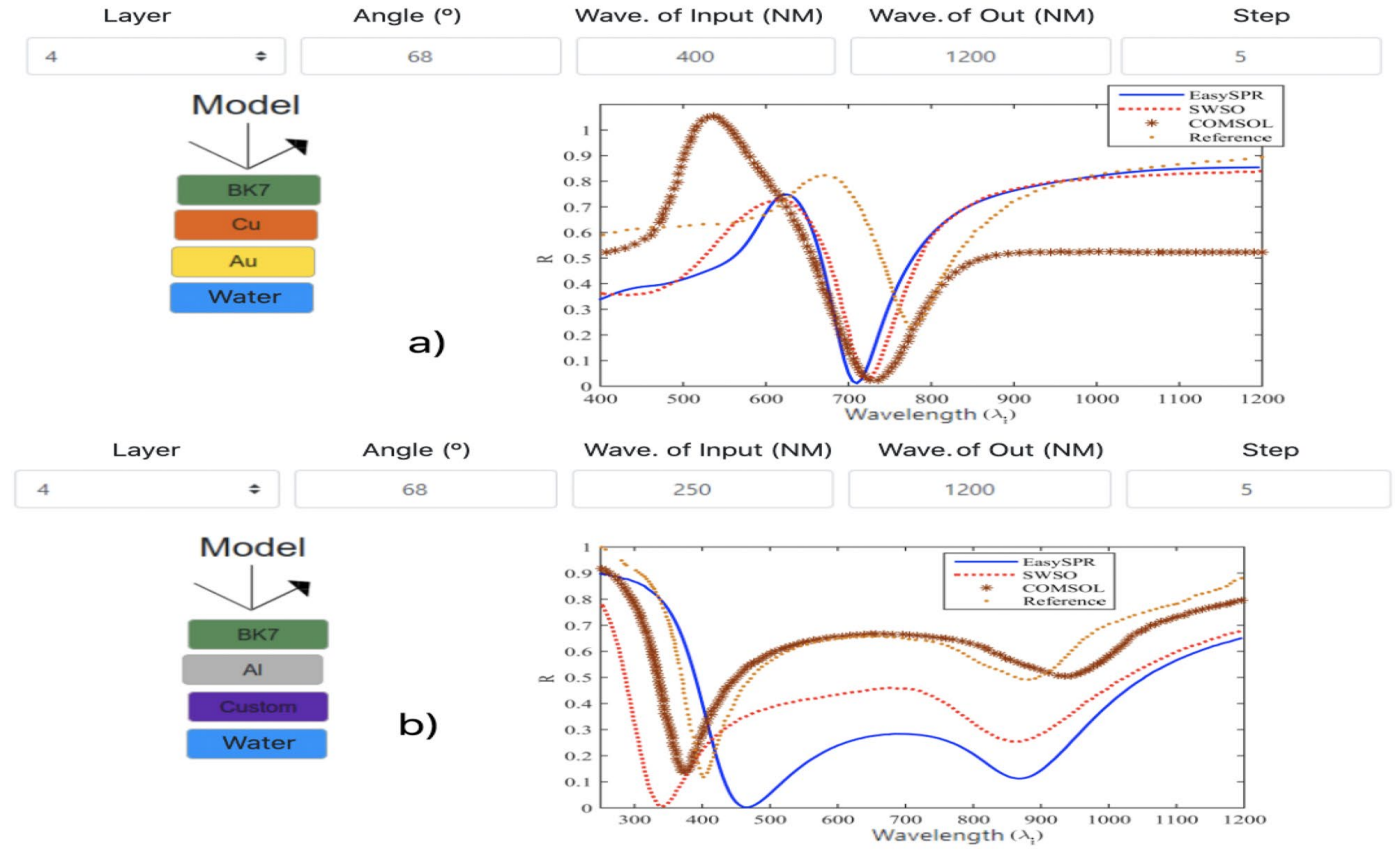
WIM SPR curves and respective models for the scenarios (**a**) 4 and (**b**) 5. Indication of the configuration parameters and responses for each simulator.

The Fig. 12 illustrated the sensor models and respective SPR curves. The simulators used in WIM scenarios present equivalent results. Once the optical parameters of the materials are wavelength dependent, the programming language and interpolation approach will affect the final result. A resonance shift is noticed on EasySPR response due the fact that aluminum $\epsilon _m(\lambda )$ in the EasySPR is based on the McPeak^48^ optical film dataset, while the other simulators are based on Rakić, Palik or Johnson experimental report^35,49,50^. However, the step parameter used into EasySPR spline interpolation, can fine-tuning with a value that archive more precisely the desired wavelength granularity, and consequently the material optical response.

## Conclusion

A novel web-based simulator for surface plasmon resonance sensor was demonstrated. With a friendly user interface, enable an agile SPR sensor modeling through drag &drop actions. The multilayer structure responsible for phenomenon excitation can be created with several materials, present in the simulator database. Due to the visual-construction strategy for model creation and visualization, users can easily performs different sensor designs. Moreover, special materials called Custom and Complex Custom were included as an option to improve simulations, combinations and lattices at water and gaseous interfaces. Four simulation options are providing by the proposed EasySPR Simulator: (1) simulate AIM, for sensor design to operating in angular mode, (2) simulate WIM, for sensors operating in spectral mode, (3) localized-SPR, for sensor with nanoparticles as metallic layer, and (4) the Sensorgram which simulate the principle of dynamic behavior for multi-substance sensing. Different scenarios of simulation demonstrate the sensor feasibility and completeness. The simulation results are displayed in terms of graphical visualization and numerical results at EasySPR, which meet the main topics for study and development of surface plasmon resonance based sensors.

## Data Availability

The data and material used in this work is available from the corresponding author upon reasonable request.
